# Stunting and lead: using causal mediation analysis to better understand how environmental lead exposure affects cognitive outcomes in children

**DOI:** 10.1186/s11689-020-09346-x

**Published:** 2020-12-16

**Authors:** Kelsey M. Gleason, Linda Valeri, Anuraj H. Shankar, John F. Obrycki, Md Omar Sharif Ibne Hasan, Golam Mostofa, Quazi Quamruzzaman, Robert O. Wright, David C. Christiani, David C. Bellinger, Maitreyi Mazumdar

**Affiliations:** 1 Department of Medicine, Robert Larner M.D. College of Medicine, Burlington, VT USA; 2 Department of Biostatistics, Columbia Mailman School of Public Health, New York, NY USA; 3 Department of Nutrition, Harvard T.H. Chan School of Public Health, Boston, MA USA; 4 Department of Neurology, Boston Children’s Hospital, 300 Longwood Avenue, Boston, MA 02115 USA; 5 Dhaka Community Hospital, Dhaka, Bangladesh; 6 Department of Preventive Medicine, Icahn School of Medicine at Mt. Sinai, New York, NY USA; 7 Department of Environmental Medicine and Public Health, Icahn School of Medicine at Mount Sinai, New York, NY USA; 8 Department of Environmental Health, Harvard T.H. Chan School of Public Health, Boston, MA USA

**Keywords:** Bangladesh, Bayley Scales of Infant Development, Cognitive development, Blood lead, Mediation analysis, Stunting

## Abstract

**Background:**

Many children in Bangladesh experience poor nutritional status and environmental lead exposure, both of which are associated with lower scores on neurodevelopmental assessments. Recent studies have suggested that part of lead’s adverse effects on neurodevelopment are caused in part by lead’s effect on growth. New statistical methods are now available to evaluate potential causal pathways in observational studies. This study used a novel statistical method to test the hypothesis that stunting, a measure of linear growth related to poor nutrition, is a mediator and/or an effect modifier of the lead exposure’s adverse effect on cognitive development.

**Methods:**

Participants were 734 children from a longitudinal birth cohort established in rural Bangladesh to study the health effects of prenatal and early childhood environmental metal exposures. Lead exposure was estimated using umbilical cord blood samples obtained at birth and blood obtained via venipuncture at age 20–40 months. Stunting was determined using the World Health Organization’s standards. Neurodevelopment was assessed at age 20–40 months years using the Bayley Scales of Infant and Toddler Development, Third Edition (BSID-III). We evaluated the effect of lead on stunting and whether the effect of lead on cognitive scores is modified by stunting status in multivariable regression analyses. We then conducted a novel 4-way mediation analysis that allows for exposure-mediator interaction to assess how much of the effect of lead on cognitive scores is explained by the pathway through stunting (mediation) and how much is explained by the interaction between lead and stunt (effect modification).

**Results:**

Stunting was not a mediator of the effect of lead in our analyses. Results suggested effect modification by stunting. In an area of Bangladesh with lower lead exposures (median umbilical cord blood lead concentration, 1.7 μg/dL), stunting modified the relationship between prenatal blood lead concentrations and cognitive score at age 2–3 years. A 1-unit increase in natural log cord blood lead concentration in the presence of stunting was associated with a 2.1-unit decrease in cognitive scores (*β* = − 2.10, SE = 0.71, *P* = 0.003). This interaction was not found in a second study site where lead exposures were higher (median umbilical cord blood lead concentration, 6.1 μg/dL, *β* = − 0.45, SE = 0.49, *P* = 0.360).

**Conclusions:**

We used a novel method of mediation analysis to test whether stunting mediated the adverse effect of prenatal lead exposure on cognitive outcomes in Bangladesh. While we did not find that stunting acted as mediator of lead’s effect on cognitive development, we found significant effect modification by stunting. Our results suggest that children with stunting are more vulnerable to the adverse effects of low-level lead exposure.

## Introduction

Environmental lead exposure is associated with a wide range of neurodevelopmental deficits in children, with no threshold below which lead does not have adverse effects [1–7] Chronic malnutrition is also associated with cognitive deficits [8–11]. Children who experience food insecurity and are subjected to environmental contaminants, such as metals, may be particularly prone to neurodevelopmental delays and other negative health outcomes [12].

One common indicator of a nutritional deficit is stunted growth, which occurs when a child’s height (or length) for a given age is below the 5^th^ percentile [13]. Current estimates show that the world’s poorest children have stunting rates more than double those of the richest [14, 15]. Similar trends are seen in the global burden of lead exposure, with children in developing countries at the greatest risk of exposure to this heavy metal [16]. Such findings highlight the strong likelihood of concurrent exposures, yet to our knowledge, only one study has investigated the joint effects and interactions of co-exposure to lead and stunting on neurodevelopment; no significant stunting-by-metal interactions for lead, arsenic, and manganese were reported [12].

Children in Bangladesh experience both stunting and exposure to metals [12, 17]. Thirty-six percent of children under the age of 5 years in Bangladesh are stunted [11], and stunting rates within the country are higher as socioeconomic status declines [17]. At the district administrative level, stunting rates range from 28 to 51%, and over half of the districts in Bangladesh (39/64) have stunting rates greater than the World Health Organization’s (WHO) critical threshold of 40% [18]. Blood metal levels, such as blood lead, are not surveyed nationally; however, previous research found that 78% of children from urban and rural areas had blood lead levels above the CDC reference level of 5 μg/dL [19] and 87% of children at a primary school in Dhaka had BLLs ≥ 10 μg/dL [20]. In contrast, approximately 97.5% of children in the USA have blood lead levels below the current reference level of 5 μg/dL [21].

Stunting may be a *mediator* of the lead exposure-cognitive development relationship, or part of the causal pathway. Many studies have shown an inverse correlation between blood lead concentration and children’s height [19, 22, 23], with postulation that lead causes decreases of gonadotropin secretion, and that abnormalities in growth hormone axis can contribute to adverse neurodevelopmental outcomes [24]. Stunting might also act as an *effect modifier* that is the adverse effect of lead is greater among children with stunting. One potential mechanism for this is increased absorption of lead: children with compromised nutritional status may absorb more metals into their bodies compared to children with adequate nutritional intake, leading to greater declines in neurodevelopment [25, 26]. Additionally, both lead and malnutrition affect neuronal metabolism. The combination could lead to greater declines in neurodevelopment. Understanding whether growth is an effect modifier or mediator is important to design effective interventions to improve neurodevelopmental outcomes.

Mediation analysis is an established method used in social and epidemiological studies to understand the impact of variables in causal pathways or biological mechanisms. New statistical methods have been developed to understand the contributions of effect modification and mediation in epidemiological studies using a causal framework [27–29]. Using mediation analysis, the effect of lead can be decomposed into the component due to only mediation through stunting (the pure indirect effect), only interaction between lead and stunting (the reference interaction), both (the mediated interaction), or neither (the controlled direct effect) [30]. Past research in this cohort has found negative effects of metals on neurodevelopment, both in individual concentrations and in mixtures [30].

The aim of this study was to evaluate the role of stunting on the relationship between lead exposure and neurodevelopment scores using a Bangladeshi birth cohort. Our previous studies from this cohort revealed an association between prenatal lead exposure and adverse neurodevelopmental outcomes in early childhood [31], a finding that is consistent with decades of research. We have also used data from this cohort to understand the effect of metal mixtures on neurodevelopment [30, 32], to apply new statistical methods for the study of multi-pollutant mixtures [33], and to validate gene-environment studies [34]. In this analysis, we hypothesize that stunting acts as a mediator of environmental lead exposure. If our hypothesis is correct, one implication is that interventions targeted to improve chronic malnutrition would be effective in reducing the adverse effects of environmental lead exposure. Our previous studies have shown lead is associated with decreased cognitive scores in children and its effect is greater than those of other environmental metals. We have also shown that when blood lead levels are high, lead is associated with decreased cognitive scores and effects of other metals are not detected [31]. Therefore, in this study, we focus on lead rather than on environmental mixtures.

## Methods

The study population has been described previously [30, 31, 35, 36]. Briefly, between 2008 and 2011, pregnant women in the Sirajdikhan (alternate spelling Serajdikhan) and Pabna upazilas (administrative regions) of Bangladesh served by Dhaka Community Hospital (DCH) were enrolled and followed through pregnancy and delivery. Children were followed at birth, age 12 months, and age 20–40 months.

The research team obtained informed consent from the parents of all children. The project was approved by the Human Research Committees at the Harvard T.H. Chan School of Public Health (HSPH) and DCH. The Committee for Clinical Investigation at Boston Children’s Hospital (BCH) ceded review of this study to HSPH.

We enrolled 1613 pregnant women in the birth cohort study. Of these, 1153 pregnancies resulted in singleton live births and 5 pregnancies resulted in twin births. Of the remaining women, 123 were lost to follow-up, 125 withdrew from study activities, 132 pregnancies resulted in miscarriage, and 75 pregnancies resulted in stillbirth. A total of 1153 women and their children participated in follow-up studies, including 815 children who participated in neurodevelopmental assessments at age 20–40 months. Complete data were available for 734 children, and this is the sample used for analysis.

### Environmental exposure assessment

Exposure to lead, arsenic, and manganese was assessed using blood samples collected at two time points: at birth using the umbilical cord blood and at 20–40 months of age collected via venipuncture, representing early childhood exposure. We collected the blood in trace metal-free tubes. The umbilical cord blood samples were analyzed at the Trace Metals Laboratory at HSPH using ICP-MS. Venous blood samples were analyzed at HSPH and the Icahn School of Medicine at Mount Sinai using similar protocols [30]. The average limit of detection (LOD) for lead and arsenic was 0.01 μg/dL; the LOD for manganese was 0.05 μg/dL.

### Stunting

Trained field staff measured the weight, length, and head circumference of the newborns and children [32]. We measured height and head circumference to the nearest 0.1 cm and weight to the nearest 0.1 kg. We calculated *z*-scores for child’s height-for-age using the WHO’s Multicentre Growth Reference Study (MGRS) [13]. These standards provide a single international standard of physiological growth for all children from birth to 5 years of age. Specific computations were performed with the WHO Anthro and Macros (Version 3.2.2). Children with height-for-age less than or equal to 2 *z*-scores below the median of the WHO’s child growth standards were classified as stunted.

### Neurodevelopmental outcomes

We translated the Bayley Scales of infant and Toddler Development™, Third Edition (BSID-III™) and adapted it for use in rural Bangladesh [37]. The BSID-III was administered by trained study personnel blinded to participants’ environmental exposure measures. The quality control included review of all BSID-III videotapes by site leaders (Ibne Hasan and Halder), frequent observed assessments (Mazumdar), and review of 5% of the videotaped administrations of the BSID-III by a senior neuropsychologist (Bellinger).

### Covariates

We collected demographic information using structured questionnaires to obtain data on age, education, tobacco exposure, socioeconomic status, and dietary information [19]. Maternal height and weight were recorded at the initial study enrollment visit during the 1st trimester of pregnancy. Maternal education was recoded and collapsed into a dichotomous variable (primary education or less/more than a primary education) due to small numbers in the higher education categories. This categorization is consistent with other studies from Bangladesh [38–40]. We used a locally validated dish-based semi-quantitative food questionnaire (FFQ) to collect dietary information [41]. A measure of maternal protein intake during pregnancy was derived from the FFQ and coded to indicate weekly protein consumption of fish, meat, and eggs as high (> 26 units), medium (12.5–26 units), or low (< 12.5 units). Interviewers also administered the Home Observation for Measurement of the Environment (HOME) Inventory [42]. This inventory was previously translated and adapted for use in Bangladesh [43]. Mothers’ IQ was estimated using Raven’s Progressive Indices [44].

### Statistical methods

We performed statistical analyses using RStudio version 1.2.5033 (using R version 4.0.1) [45] and Stata version 16 [46]. We examined distributional plots and descriptive statistics for all variables and performed additional analyses stratified by clinic site. We evaluated for potential selection bias by comparing baseline characteristics of the birth cohort to those of the study sample. We modeled blood metal concentrations as natural log-transformed continuous variables. This transformation achieves a common scale and accounts for the right-skewedness of the biomarker levels. One study ID had a blood sample below the LOD for venipuncture lead, arsenic, and manganese at the 20–40 months of visit. We used one half of the LOD for this observation. Cognitive scores were approximately normally distributed and were modeled as continuous outcomes.

### Mediation analysis

**Fig. 1:**
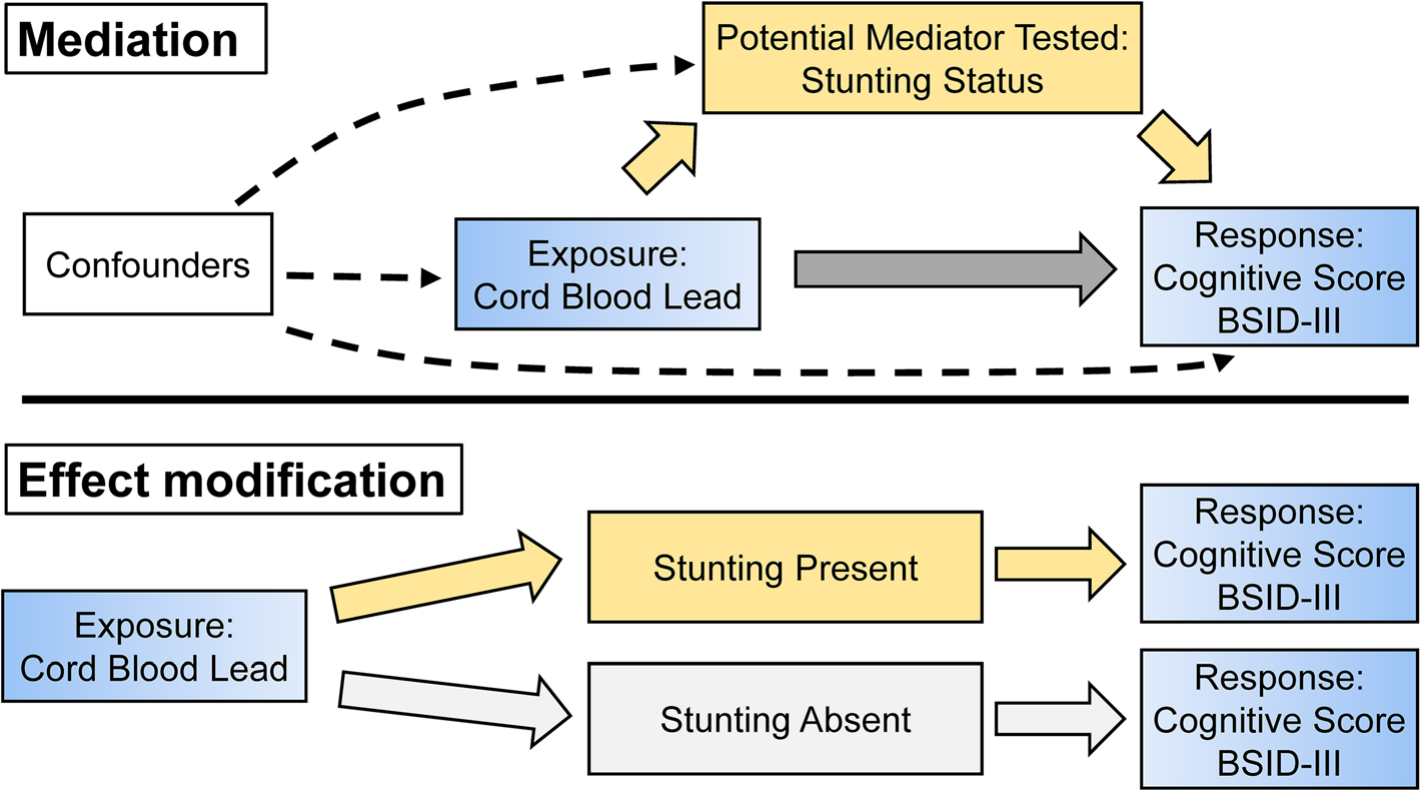
Diagram showing the mediation model components testing the effect of cord blood lead on cognitive score with stunting status as a mediator, following a previous directed acyclic graph [28]

We conducted mediation analysis to evaluate whether stunting was acting as a mediator of the relationship between cord blood lead concentration and cognitive test scores in early childhood using the 4-way decomposition proposed by VanderWeele (Fig. 1). The mediation analysis was conducted using the med4way command in Stata [28, 32], a package that our group developed previously for mediation analysis. For this specific study, we tested the effect of a change in cord blood lead from the 25th to the 75th percentile. As stunting was a dichotomous variable, the parameter *m*, denoting the level of the mediator, was set to 0 to estimate the controlled direct effect (the effect of lead that is neither due to mediation nor interaction). The analyses require adjustment for exposure-outcome, exposure-mediator, mediator-outcome confounders. All other covariates were held at their respective means. Using this mediation analysis, the effect of lead can be decomposed into the component due to only mediation through stunting (the pure indirect effect), only interaction between lead and stunting (the reference interaction), both (the mediated interaction), or neither (the controlled direct effect) [30]. Mean, median, and confidence interval estimates for each component of the decomposition were obtained for each site.

The covariates used in the model were selected to meet the criteria for using the mediation analysis [28], including all confounders of exposure-mediator, exposure-outcome, and mediator-outcome are adjusted for in the model. Additionally, there must be no mediator-outcome confounders affected by the exposure. Unadjusted models were analyzed for each of these components and possible confounders were added to a subsequent adjusted model. New adjusted models were created for each variable of interest. We assumed a variable was a confounder is the effect estimate for the predictor changed by greater than 10%.

### Regression models and interaction analyses

Mediation analysis requires the specification of a model for the outcome (cognitive score) and a model for the mediator (stunting). A multivariable linear regression model was used to model the mean of cognitive score conditionally on cord blood lead, stunting, and confounders. A logistic regression model was used to model the conditional odds of stunting score given cord blood lead and confounders. The following covariates were included in models to evaluate for confounding the study site, child’s sex, child’s birth length, child birth weight, child’s age at the time of neurodevelopmental testing, mother’s age at the time of the child’s birth, maternal education, maternal IQ, maternal height and weight, HOME score, secondhand smoke exposure, maternal protein intake, cord blood arsenic and manganese, and early childhood blood metal concentrations. To identify if squared terms were needed in the regression model, we used the generalized additive model (gam) spline function in R’s mgcv package (Mixed GAM computation vehicle) [47]. The criteria for remaining in the model consisted of a *p* value less than 0.05 and an effective degrees of freedom (EDF) greater than 1. Given the important differences in exposure profiles and baseline characteristics observed across the two study sites, regression models were stratified by clinic in primary analyses and adjusted for in secondary analyses. We evaluated for effect modification by stunting status using interaction terms in the regression models. We performed additional analyses using the *z*-score length-for-age as a continuous variable for child growth.

## Results

### Study population

A summary of the study population is presented in Table 1. In general, the population was primarily agricultural/rural with low rates of formal education for mothers. There were important differences between the sites. Children from Sirajdikhan had higher blood lead concentrations in cord blood samples (median 6.1 vs. 1.7 μg/dL) and at 20–40 months (median 8.2 vs. 4.0 μg/dL) than children from Pabna. Children from Sirajdikhan also had higher rates of stunting than children from Pabna (66% vs. 40%), although the mothers in Sirjadikhan were generally heavier (median 47 vs. 45 kg). Mothers in Pabna were more likely to consume high amounts of protein during pregnancy compared to mothers in Sirajdikhan (44% vs 3%). We did not find significant differences in baseline characteristics between the full original cohort and the complete case study sample used in this analysis.

**Table 1:** Study population

Characteristic	Pabna (*n* = 351)			Sirajdikhan (*n* = 383)		
	*n (%)*	Mean ± SD	Range	*n (%)*	Mean ± SD	Range
Child characteristics						
Female sex	173 (49%)			189 (49%)		
Birth Weight (kg)	351	2.8 ± 0.5	0.8–4.5	383	2.9 ± 0.3	1.0–3.5
Birth Length (cm)	351	46.9 ± 2.4	33.0–56.0	383	46.1 ± 2.6	34.0–74.0
Age at study visit (months)	351	28.0 ± 2.8	19.9–40.3	383	28.2 ± 2.9	22.5–36.1
*Z*-score length-for-age	351	(− 1.8) ± 1.0	(− 6.9)–0.8	383	(− 2.3) ± 1.1	(− 12.1)–1.1
Stunted	351	142 (40%)		253 (66%)		
Maternal characteristics						
Age at childbirth (years)	351	22.9 ± 4.1	18.0–41.0	383	23.0 ± 4.4	18.0–40.0
Height (cm)	351	151.0 ± 5.1	134.0–168.0	383	151.4 ± 6.2	135.0–189.0
Weight (kg)	351	45.1 ± 7.0	30.0–70.0	383	47.9 ± 8.2	31.0–80.0
Protein intake, pregnancy						
Low (< 12.5 units/week)	1 (0.3%)			195 (51%)		
Medium (12.5–26 units/week)	196 (56%)			177 (46%)		
High (> 26 units/week)	154 (44%)			11 (3%)		
Education						
Primary education or less	160 (46%)			185 (48%)		
Secondary education or more	191 (54%)			198 (52%)		
Any smokers in household: yes	177 (50%)			130 (34%)		
Prenatal metal exposures						
Cord blood As (μg/dL)	351	1.5 ± 2.2	0.1–27.7 (IQR 0.6, 1.6)	383	0.5 ± 0.6	0.1–7.4 (IQR 0.3, 0.6)
Cord blood Mn (μg/dL)	351	18 ± 30	1.7–303.2 (IQR 4.9, 18.0)	383	6.5 ± 7.6	1.2–88.6 (IQR 3.9, 6.6)
Cord blood Pb (μg/dL)	351	2.7 ± 6.9	0.3–79.2 (IQR 1.2, 2.4)	383	7.3 ± 4.6	1.0–36.0 (IQR 4.0, 9.7)
Child metal exposures						
Venous blood As (μg/dL)	351	1.0 ± 0.8	0.1–5.7 (IQR 0.4, 1.4)	383	0.9 ± 0.7	LOD^a^–3.1 (IQR 0.2, 1.3)
Venous blood Mn (μg/dL)	351	2.1 ± 2.1	0.6–36.5 (IQR 1.4, 2.4)	383	2.2 ± 1.0	LOD^b^–11.7 (IQR 1.6, 2.6)
Venous blood Pb (μg/dL)	351	4.5 ± 2.1	1.1–18.0 (IQR 3.0, 5.2)	383	9.1 ± 4.5	LOD^a^–39.9 (IQR 5.9, 11.2)

Note: The dataset used in this study was a complete cases dataset, so each variable was reported for every study participant

^a^The limit of detection was 0.010 μg/dL

^b^The limit of detection was 0.050 μg/dL

### Mediation analysis

We performed stratified analyses by site due to concerns about residual confounding, a strategy that is consistent with other reports from this cohort [31, 48]. A table reporting the 4 decomposed effects is shown in Table 2. In Pabna, where lead exposure was generally lower, the mediation analysis demonstrated a significant effect resulting from the reference interaction (the effect due only to interaction) between cord blood lead concentration and stunting, which reduced cognitive scores − 0.779 (*p* = 0.001). We did not find evidence of a reference interaction in Sirajdikhan (*p* = 0.483). No significant effect was found for mediation only, mediation and interaction combined, or neither mediation nor interaction in either site. Collectively, these results suggest the presence of effect modification by stunting in an area with low-level lead exposure.

**Table 2:** Mediation analysis of the effect of log cord blood lead concentrations on cognitive scores when mediated by stunting status at both study sites

Mediation Analysis Effects	Coefficient	95% confidence interval		***p*** value
		***Lower bound***	***Upper bound***	
*Pabna*				
Total effect	− 0.160	− 0.787	0.467	0.617
Controlled direct effect	0.560	− 0.169	1.289	0.132
Reference interaction	− 0.779	− 1.248	− 0.310	0.001
Mediation interaction	0.128	− 0.033	0.289	0.120
Pure indirect effect	− 0.069	− 0.182	0.044	0.230
Proportion mediated	− 0.368	− 1.922	1.186	0.642
Proportion attributable to interaction	4.067	− 11.814	19.947	0.616
*Sirajdikhan*				
Total effect	0.001	− 0.460	0.463	0.996
Controlled direct effect	0.205	− 0.507	0.918	0.573
Reference interaction	− 0.209	− 0.792	0.374	0.483
Mediation interaction	0.002	− 0.023	0.028	0.850
Pure indirect effect	0.002	− 0.023	0.027	0.849
Proportion mediated	4.622	− 2022.850	2032.095	0.996
Proportion attributable to interaction	− 196.420	− 86513.200	86120.360	0.996

Analysis conducted using med4way command in Stata version 16; for Sirajdikhan, a(0) was set to 1.381, the 25th percentile of log cord blood lead μg/dL; a(1) was set to 2.268, the 75th percentile of log cord blood lead μg/dL; and *m* was set to 0; for Pabna, a(0) was set to 0.149, the 25th percentile of log cord blood lead μg/dL; and a(1) was set to 0.895, the 75th percentile of log cord blood lead μg/dL. Other included covariates were sex of child, child’s age in months, mother’s approximate age in years, education category, IQ, HOME score, environmental smoke exposure, protein intake, child’s birth length, child’s birth weight, mother’s weight, mother’s height, log cord blood arsenic and manganese, log blood lead, arsenic, and manganese collected at 20–40 months

### Effect modification

In our regression models, we confirmed a significant interaction between lead and stunting status, both in the cohort as a whole and in our stratified analyses. In the 3-way model for the entire cohort, study site had a main effect on cognitive scores (*β* = − 2.77, SE = 1.15, *p* = 0.016) with Sirajdikhan coded as study site 0 and Pabna coded as study site 1. The results for the 3-way interaction confirmed a significant interaction for log cord blood lead (μg/dL)*stunting*study site (*β* = − 1.67, SE = 0.89, *p* = 0.06) on cognitive scores. In our stratified models, in Pabna, an area where lead levels were low, we found a 1-unit increase in natural log cord blood lead μg/dL in the presence of stunting was associated with a 2.10 decrease in cognitive scores (*p* = 0.003, standard error = 0.713). In Sirajdikhan, where blood lead concentrations were generally higher, this interaction was not found (Table 3). In sensitivity analysis, we found a significant interaction of log cord blood lead (μg/dL)*z-score height-for-age in Pabna (*β* = 0.86, SE = 0.39, *p* = 0.03) and results that approached statistical significance in Sirajdikhan (*β* = 0.42, SE = 0.22, *p* = 0.06) (Table 3). These findings confirm our hypothesis that child growth is an important effect modifier of lead.

**Table 3:** Regression results for the effect of cord blood lead concentration and childhood stunting on cognitive neurodevelopment at study sites

Model component	Pabna (***n*** = 351)		Sirajdikhan (***n*** = 383)	
	***β (Std error)***	***p value***	***β (Std error)***	***p value***
*Key predictors and interaction*				
Log cord blood lead (μg/dL)	0.774 (0.497)	0.120	0.317 (0.417)	0.448
Stunted	1.020 (0.652)	0.119	0.339 (0.938)	0.718
Log cord blood lead μg/dL*Stunted	− 2.100 (0.713)	0.003	− 0.451 (0.492)	0.360
*Model covariates*				
Child characteristics				
Child sex	− 0.200 (0.469)	0.670	− 0.223 (0.304)	0.464
Birth weight (kg)	− 0.335 (0.589)	0.570	1.779 (0.552)	0.001
Birth length (cm)	1.255 (1.292)	0.332	0.658 (0.431)	0.128
Birth length (cm)^2^	− 0.008 (0.014)	0.580	− 0.007 (0.004)	0.114
Age at study visit (months)	6.269 (1.039)	4.3*10^-9^	4.359 (1.112)	1.1*10^-4^
Age at study visit (months)^2^	− 0.089 (0.017)	5.3*10^-7^	− 0.066 (0.019)	7.7*10^-4^
Mother characteristics				
Age at childbirth (years)	0.011 (0.061)	0.854	0.010 (0.037)	0.792
Height (cm)	− 0.068 (0.052)	0.194	0.012 (0.026)	0.637
Weight (kg)	− 0.011 (0.038)	0.777	− 0.014 (0.021)	0.500
Protein intake, pregnancy	0.771 (0.491)	0.118	− 0.424 (0.325)	0.192
Raven IQ score	0.014 (0.045)	0.759	− 0.019 (0.020)	0.334
Education	1.300 (0.513)	0.012	0.305 (0.321)	0.342
Any smokers in household	− 0.258 (0.462)	0.578	0.035 (0.316)	0.911
Composite HOME Score	0.043 (0.098)	0.664	0.248 (0.086)	0.004
Prenatal metal exposures				
Log cord blood As (μg/dL)	0.743 (0.368)	0.044	− 0.252 (0.260)	0.334
Log cord blood Mn (μg/dL)	− 0.421 (0.347)	0.226	0.097 (0.332)	0.770
Child metal exposures				
Venous blood As (μg/dL)	− 0.781 (0.475)	0.102	0.768 (0.303)	0.012
Venous blood As (μg/dL)^2^	0.018 (0.310)	0.954	− 0.054 (0.136)	0.694
Venous blood Mn (μg/dL)	− 1.231 (0.624)	0.049	− 0.415 (0.428)	0.332
Venous blood Pb (μg/dL)	0.191 (0.581)	0.743	− 0.721 (0.325)	0.027
Intercept	− 79.714 (34.690)	0.022	− 40.453 (19.954)	0.043

The variables used in these separate models (one for each site) were as follows: All variables in the table were continuous except the following: stunted (categorical, 0 and 1 with 1 indicating stunting), child sex (categorical male =1, female = 2), protein intake during pregnancy (categorical, with low = 1, medium = 2, and high = 3), education (categorical, with 0 = primary education or less, and 1 = secondary education or more), and any smokers in the household (categorical, with 0 = No, and 1 = Yes)

## Discussion

In this study, we applied causal mediation analysis to assess the contribution of stunting, a measure of impaired growth related to poor nutrition, to the adverse neurodevelopmental outcomes associated with environmental lead exposure. We found that stunting modifies the adverse effects of lead, such that the adverse effect of lead was greater among children who were stunted. Effect modification was most apparent among children with low-level lead exposure. The results suggest that a child experiencing stunting with a log-cord blood lead concentration of 4 μg/dL may have cognitive scores 8.4 points lower than a child with similar cord blood lead exposure but without stunting. Our analyses did not reveal evidence of mediation, suggesting that stunting itself it does not lie within the causal pathway between lead exposure and adverse cognitive development.

Dietary interventions have long been part of a comprehensive strategy to prevent and mitigate the neurodevelopmental effects of environmental lead exposure in children [49]. These strategies are based on observations that blood lead is associated with lower intake of iron [50], calcium [51], vitamin C [52], and D[53] and that blood lead concentration among children is associated decreased height of children [22]. These observational studies are limited in determining causality and in answering important questions needed to design effective interventions [54, 55].

In this study, we apply a novel statistical method that simultaneously evaluates for mediation and effect modification [27, 28]. In our example, while we observe that stunted children are more vulnerable to the adverse effects of low-level, we also determine that stunting is unlikely to be part of the mechanism by which lead causes adverse cognitive development. These results suggest that resources might best be used to screen for lead exposure among populations with high rates of stunting and reduce lead exposure in these populations.

### Strengths and limitations

Our studies used biomarkers of metal exposure using ICP-MS, a gold standard for the measure of blood lead concentration and a measure not widely available in low-resource settings such as Bangladesh. In addition to lead, we were able to control for exposure to other toxic metals in our models, which is an additional strength. Understanding the effects of metal mixtures was not the focus of this analysis. Additionally, we were able to adjust for a large number of potential confounders as well as predictors of stunting and neurodevelopment. However, we recognized the potential for unmeasured confounding variables and stratified analyses by clinic reduced the heterogeneity in demographics and potential for residual confounding.

## Conclusion

Causal mediation analysis enables investigation of potential mechanisms of disease in epidemiological studies. We used a novel method of mediation analysis to test whether stunting, a measure height-for-age related to poor nutrition, mediated the adverse effect of prenatal lead exposure on cognitive outcomes in Bangladesh. While we did not find that stunting acted as mediator, we found significant effect modification by stunting. Our study suggests that stunted children are more vulnerable to the adverse effects of low-level lead exposure, interventions that include targeting populations with high rates of stunting for lead screening and reduction of lead exposure are most likely to be effective in improving neurodevelopmental outcomes.

## Data Availability

The dataset used and/or analyzed during the current study are available from David Christiani, Harvard T.H. Chan School of Public Health, dchris@hsph.harvard.edu.

## Abbreviations

BCH: Boston Children’s Hospital
HSPH: Harvard T.H. Chan School of Public Health
BSID-III: Bayley Scales of Infant and Toddler Development, Third Edition
DCH: Dhaka Community Hospital
FFQ: Food Frequency Questionnaire
HOME: Home Observation for Measurement of the Environment Inventory
LMIC: Low- to middle-income country
LOD: Limit of detection
mgcv: Mixed GAM Computation Vehicle
MGRS: Multicentre Growth Reference Study
SD: Standard deviation
WHO: World Health Organization
